# The flavivirus polymerase NS5 regulates translation of viral genomic RNA

**DOI:** 10.1093/nar/gkaa242

**Published:** 2020-04-20

**Authors:** Teodoro Fajardo, Thomas J Sanford, Harriet V Mears, Annika Jasper, Skye Storrie, Daniel S Mansur, Trevor R Sweeney

**Affiliations:** 1 Division of Virology, Department of Pathology, University of Cambridge, Addenbrooke's Hospital, Hills Road, Cambridge, UK; 2 Laboratory of Immunobiology, Department of Microbiology, Immunology and Parasitology, Universidade Federal de Santa Catarina, Florianópolis, Brazil

## Abstract

Flaviviruses, including dengue virus and Zika virus, contain a single-stranded positive sense RNA genome that encodes viral proteins essential for replication and also serves as the template for new genome synthesis. As these processes move in opposite directions along the genome, translation must be inhibited at a defined point following infection to clear the template of ribosomes to allow efficient replication. Here, we demonstrate *in vitro* and in cell-based assays that the viral RNA polymerase, NS5, inhibits translation of the viral genome. By reconstituting translation *in vitro* using highly purified components, we show that this translation block occurs at the initiation stage and that translation inhibition depends on NS5-RNA interaction, primarily through association with the 5′ replication promoter region. This work supports a model whereby expression of a viral protein signals successful translation of the infecting genome, prompting a switch to a ribosome depleted replication-competent form.

## INTRODUCTION

Members of the *Flaviviridae*, including dengue virus (DENV), Zika virus (ZIKV), West Nile virus (WNV) and yellow fever virus are some of the most important emerging and re-emerging pathogens (1–3). Habitat range expansion of their mosquito vectors has resulted in increasing human populations at risk of infection. Although typically self-limiting, flavivirus infection can cause fatal complications such as haemorrhagic fever, while ZIKV infection in pregnant women has been linked with neonatal microcephaly (4).

**Figure 1.**
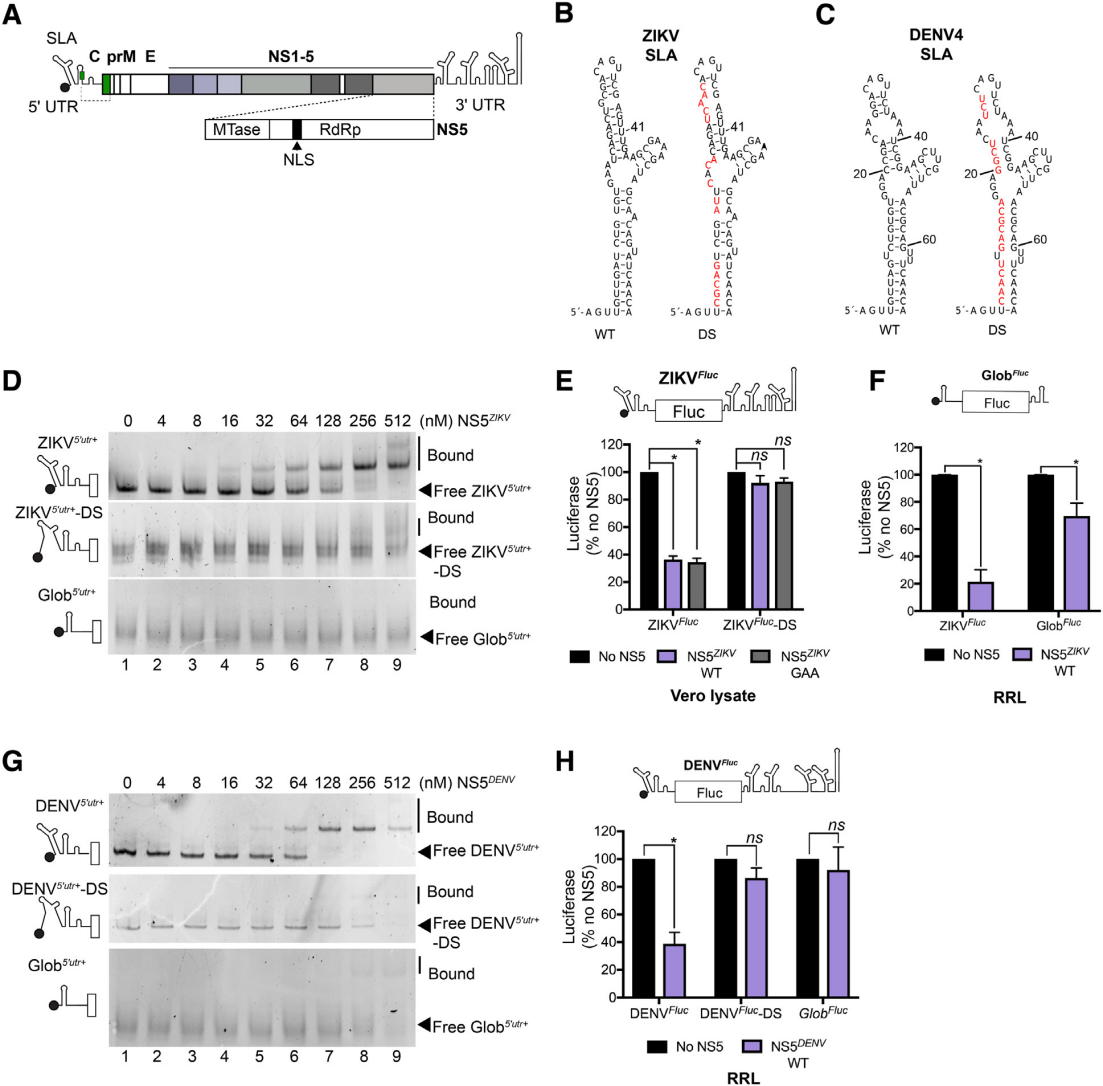
NS5 inhibits translation. (**A**) Schematic of the ZIKV genome. The start codon of the long open reading frame is a green box. C, capsid sequence, partially shown as a solid line to show conserved RNA structure, and in green, full capsid sequence represented by the dotted line. prM, precursor membrane; E, envelope; NS, non-structural; SLA, stem-loop A; nuclear localization sequence (NLS). (**B**) Nucleotide sequence of ZIKV PE243 SLA. SLA was mutated to destabilize (DS) SLA by replacing the wildtype sequence with the red nucleotides shown in the right panel. (**C**) Nucleotide sequence of DENV4 SLA. Mutations introduced to destabilize (DS) DENV4 SLA are indicated in red in the right panel. (**D**) EMSA of the first 359 nt of wildtype (ZIKV*^5^′^utr+^;* upper panel) or SLA-destabilized (ZIKV*^5^′^utr+^*-DS; middle panel) ZIKV RNA or Glob*^5^′^utr+^* RNA (all shown schematically on left) with increasing concentrations of NS5*^ZIKV^*. Free and bound RNA are indicated. (**E**) Luciferase production after incubation of ZIKV*^Fluc^* (schematic on top) RNA in Vero lysate in the absence or presence of 256 nM NS5*^ZIKV^*. (**F**) Luciferase production after incubation of ZIKV*^Fluc^* RNA or Glob*^Fluc^* RNA (schematic on top) in the absence or presence of 256 nM NS5*^ZIKV^* in rabbit reticulocyte lysate (RRL). (**G**) EMSA of the first 400 nt of wildtype (DENV*^5^′^utr+^;* upper panel) or SLA-destabilized (DENV*^5^′^utr+^*-DS; middle panel) DENV4 RNA or Glob*^5^′^utr+^* RNA (all shown schematically on left) with increasing concentrations of NS5*^DENV^*. Free and bound RNA are indicated. (**H**) Luciferase production after incubation of DENV*^Fluc^* RNA (schematic on top) or Glob*^Fluc^* RNA in the absence or presence of 256 nM NS5*^DENV^* in RRL. (E, F and H) Values, normalized to the no polymerase control, are mean +/− SEM from three independent experiments. Statistical significance was determined by a Student's unpaired t-test with significant values indicated with an asterisk. *P<0.05. **P<0.001.

Flaviviruses possess single-stranded positive sense RNA genomes, ∼11 kb in length, that have a 5′ cap structure similar to cellular mRNAs but lack a 3′ polyA tail (5). A long open reading frame, flanked by highly structured 5′ and 3′ untranslated regions (UTR), encodes structural proteins required for virus assembly and non-structural proteins involved in replication of the viral genome (Figure 1A). Following release into the cytoplasm the genome is translated as a long polyprotein that is cleaved by viral and cellular proteases to produce mature viral proteins. The viral genome is then copied in a 3′-5′ direction by the virally encoded RNA-dependent RNA polymerase (RdRp). This negative-sense strand, in turn, serves as a template for new positive-strand synthesis for further translation or packaging into progeny virions. As translation and replication inherently move in opposite directions, viral genome usage must be tightly controlled. However, the mechanism of so-called ‘lifestyle-switching’ between translation and replication of the flaviviral genome is not well understood.

The flavivirus non-structural protein 5 (NS5) is the largest mature viral protein expressed during infection (Figure 1A). It harbours an N-terminal methyltransferase (MTase) domain responsible for generating the cap1 structure at the 5′ end of newly synthesized viral genomes which promotes translation (6) and shields viral RNA from immune detection (7,8). The MTase domain is separated by a short linker from a C-terminal RdRp domain that adopts a characteristic right-handed fingers-palm-thumb fold (9–13). NS5 binds to a conserved RNA structural feature, stem loop A (SLA), at the 5′ extremity of the flaviviral genome (14–16) which serves as a promoter for viral replication (14). Flavivirus genome replication critically depends on hybridization between complementary RNA sequences in the 5′ and 3′ ends (6,17–20). Hybridization of these sequences, termed the upstream of AUG region (UAR), the downstream of AUG region (DAR) and cyclization sequence (CS), is thought to facilitate transfer of NS5 bound to SLA to the 3′ end of the genome to initiate replication (14,21). Recently, we demonstrated that the large-scale structural rearrangements around the translation start site that accompany 5′-3′ hybridization disrupt flavivirus genome translation (6), promoting a replication-competent state.

Aside from its main roles as the viral RdRp and MTase, the flavivirus NS5 protein has been ascribed other functions that promote viral replication. The best characterized of these is the role of NS5 in disrupting host immune signalling pathways (reviewed in 22). DENV and ZIKV NS5 both target signal-transducer and activator of transcription (STAT)2 for proteasomal degradation (23–25), disrupting interferon signalling and stalling the cellular antiviral response. NS5 from different flaviviruses has also been reported to enter the nucleus due to the presence of a well conserved nuclear localization sequence (NLS) (26–29). Although the role and importance of nuclear localization remains to be clarified (30), DENV NS5 was reported to interact with components of the U5 snRNP complex and interfere with splicing during infection (31).

Here, we investigated the interplay between the NS5/SLA interaction and flaviviral genome translation. Using *in vitro* and cell-based translation experiments, we demonstrate that NS5 binding to SLA directly blocks translation of the viral genome. This inhibition occurs at the translation initiation stage as determined by detailed *in vitro* reconstitution analysis. Our findings support a model whereby, following initial rounds of translation, recruitment of newly synthesized NS5 to SLA inhibits viral protein synthesis, priming the viral RNA for replication.

## MATERIAL AND METHODS

### Plasmids and reagents

A pCC1BAC vector containing the open reading frame of the ZIKV BeH819015 isolate flanked by the 5′ and 3′ UTRs of the ZIKV PE243 isolate and bearing an inline duplicate copy of the capsid protein fused to a Nluc or mCherry gene and 2A peptide sequence (32) were kindly provided by Andres Merits. The existing SP6 promotor in the Nluc containing plasmid was replaced with a T7 promoter as previously described (6). A plasmid containing the first 359 nt of ZIKV PE243 isolate (ZIKV*^5^′^utr+^*) was previously described (6). A plasmid expressing Fluc flanked by the 5′ and 3′ regions of ZIKV PE243 isolate (ZIKV*^Fluc^*) was previously described (33). ZIKV RNA expressing plasmids with mutations in SLA (ZIKV*^5^′^utr+^*-DS, ZIKV*^Fluc^*-DS) were generated by site directed mutagenesis. Gene fragments containing the first 400 nt of DENV4 (DENV*^5^′^utr+^*) or Fluc flanked by the first 148 nt and complete 3′ UTR of DENV4 (DENV*^Fluc^*) bearing a 5′ T7 promoter and EcoRI and XhoI sites were synthesized by Integrated DNA Technologies (IDT) and cloned into pUC57. GeneBank number FJ196850 was used for the DENV4 sequence. DENV RNA expressing plasmids containing mutations in SLA (DENV*^5^′^utr^*-DS, DENV*^Fluc^*-DS) were also synthesized by IDT and cloned as described for the wild type sequences. A plasmid containing Fluc flanked by the 5′ and 3′ UTRs of human globin was previously described (8). A plasmid containing the 5′ and 3′ regions of ZIKV PE243 joined by a short linker sequence (ZIKV*^mini^*) and a mutant version in which the 3′ UAR sequence was replaced with the 5′ UAR sequence (ZIKV*^mini^*- Δ3′ UAR) were previously described (6). The tRNA_i_^Met^ transcription vector has been described (34). Recombinant human eIF expression plasmids have been described previously: eIF1 and eIF1A (35), eIF4A and eIF4B (36) and methionyl-tRNA synthetase (37). Wildtype and GAA mutant ZIKV NS5 sequences were amplified by PCR from the full-length ZIKV or full-length ZIKV-NS5-GAA plasmids previously described (6). The full length or amino acids 270–900 comprising the polymerase domain of ZIKV NS5 coding region was subcloned into pET28b (Novagen) between NheI and HindIII to generate a bacterial expression construct for NS5*^ZIKV^* with an N-terminal His_6_-tag or into pcDNA 3.1+ (ThermoFisher) between NheI and NotI with an N-terminal FLAG tag for mammalian cell expression. A gene fragment encoding NS5 from DENV4 (GeneBank number FJ196850), codon optimized for expression in E. coli was synthesized by IDT and cloned into pET28b between NheI and HindIII to generate a bacterial expression construct for NS5*^DENV^* with an N-terminal His_6_-tag. Hippuristanol was generously shared by Jerry Pelletier.

### *In vitro* transcription

Plasmids were linearized with HindIII (ZIKV*^5^′^utr+^*, ZIKV*^Fluc^* and ZIKV*^mini^*), EcoRV (DENV*^5^′^utr+^* and DENV*^Fluc^*), AgeI (ZIKV*^Nluc^*, ZIKV*^mCherry^*), FspI (Glob*^Fluc^*) or BstN1 (tRNA_i_^Met^). The globin 5′ UTR and first 97 nt of Fluc with a T7 promoter sequence were amplified from Glob*^Fluc^* by PCR to generate a double stranded template to transcribe Glob*^5^′^utr+^* RNA. ZIKV*^mCherry^* RNA was transcribed using the SP6 RiboMax transcription kit (Promega). All other RNAs were transcribed with recombinant T7 polymerase (50 ng/μl) in buffer containing 40 mM HEPES pH 7.5, 32 mM MgOAc, 40 mM DTT, 2 mM Spermidine, 10 mM each NTP and 0.2 U/μl RNaseOUT (Invitrogen) for 2 h at 37°C. ZIKV*^Nluc^* and ZIKV*^mCherry^* RNA were purified using TRI Reagent (Sigma) before ethanol precipitation. All other transcription reactions were treated with DNaseI and RNA was extracted with acidic phenol/chloroform and ethanol precipitated. Residual nucleotides were removed with Illustra MicroSpin G-50 columns (GE Healthcare). RNA was capped using the ScriptCap system (CellScript).

### Bacterial protein expression and purification

Recombinant His-tagged NS5*^ZIKV^* and NS5*^DENV^* were expressed in Rosetta 2 (DE3) pLysS *Escherichia coli* (Novagen). Cells were grown to an OD_600_ of 0.6 in 2 × TY media at 37°C. Expression was induced by adding 0.5 mM isopropyl β-d-1-thiogalactopyranoside. The induced culture was incubated at 20°C for 16 h. Cells were harvested and lysed in a buffer containing 20 mM Tris pH 7.5, 400 mM KCl, 5% glycerol, 1 mM DTT and 0.5 mM phenylmethylsulfonyl fluoride, 0.5 mg/ml lysozyme (from hen egg) and 20 mM imidazol. His-tagged proteins were isolated by affinity chromatography on Ni-NTA Agarose beads (Qiagen) and additionally purified by FPLC on a Superdex 200 Increase 10/300 GL size exclusion column (GE Healthcare) in 20 mM Tris pH 7.5, 300 mM KCl, 5% glycerol and 1 mM DTT.

### Vero lysate preparation

Vero cell lysate were prepared as described (38) with some minor modifications. Briefly, Vero cells (ATCC) were grown in T150 flasks until 80% confluency, harvested and washed twice with PBS and a short wash with a hypotonic lysis buffer (10 mM HEPES, pH 7.5, 10 mM KOAc, 0.5 mM MgOAc, 5 mM DTT and EDTA free protease inhibitor cocktail (ROCHE)). The cells were subsequently lysed in 2 ml lysis buffer by mechanically passaging in a 27G needle on ice at least 20 times. The lysate was cleared by centrifugation at 10 000 × g for 5 min. 0.75 mM CaCl_2_ and 15 U/ml of micrococcal nuclease was added for 10 min at 25°C before 3 mM EGTA was added to inhibit nuclease activity. The final lysate was stored at −80°C.

### *In vitro* translation and luciferase measurement

*In vitro* translation was performed using the Flexi RRL System (Promega). For Vero cell lysate translation reactions, 5 μl of Vero lysate prepared as in (38) was supplemented with 1.6 mM HEPES, pH7.6, 2 mM creatine phosphate, 0.01 μg μl^−1^ creatine kinase, 0.01 mM spermidine, 100 mM KCl, 2 mM MgOAc, 4 mM DTT, 25 μM amino acids (Promega). After 90 min at 30°C, reactions were terminated by addition of 50 volumes of passive lysis buffer (Promega) before luciferase signal was measured by GloMax (Promega). Luciferase values were normalized to the no NS5 control for each experiment.

### 48S complex assembly

*In vitro* reconstitution of 48S complex assembly was performed as previously described (6). 0.2 pmol RNA was incubated with the indicated eIFs (2 pmol 40S subunit, 4 pmol Met-tRNA_i_^Met^, 4 pmol eIF2, 3 pmol eIF3, 10 pmol eIF4A, 5 pmol eIF4B, 2.5 pmol eIF4F, 10 pmol eIF1, 10 pmol eIF1A) at 37°C for 10 min in a reaction volume of 20 μl Buffer A (20 mM Tris pH 7.5, 100 mM KCl, 2.5 mM MgCl_2_, 2 mM DTT, 0.25 mM spermidine, 1.6 U/μl RNaseOUT (Invitrogen), 0.4 mM guanosine triphosphate (GTP) and 2 mM adenosine triphosphate (ATP)). Purified NS5*^ZIKV^* was included at the concentrations described in the figure legends. Assembled complexes were analysed by primer extension inhibition using 2.5 U avian myeloblastosis virus reverse transcriptase (Promega) in the presence of ^32^P-labelled primer, 8 mM MgCl_2_ and 0.5 mM dNTPs. cDNA products were phenol/chloroform extracted and ethanol precipitated before being resolved on denaturing 6% polyacrylamide sequencing gels and detected by autoradiography using an FLA7000 Typhoon scanner (GE).

### Electrophoresis mobility shift assay

*In vitro* transcribed capped RNA (8.6 pmol) was heated at 75°C and snap cooled on ice in the presence of RNA refolding buffer (50 mM Tris, pH 7.5, 100 mM KCl and 5 mM MgCl_2_). RNA (860 fmol) was subsequently incubated with the indicated proteins (at a final concentration range of 0 nM to 512 nM) at 30°C for 15 min in a reaction volume of 10 μl containing Buffer A supplemented with 5 μg BSA (Sigma) and 2 μg yeast tRNA (Ambion). Reaction mixtures were preincubated at 30°C for 5 min prior to RNA addition. Following addition of 10× native RNA loading dye (0.05% bromophenol blue, 0.05% xylene cyanol FF, 50% glycerol), reactions were analysed by native PAGE on 0.5× TBE, 5% polyacrylamide gels containing 5% glycerol on ice at 4°C. Gels were stained with 1 μg/ml ethidium bromide in 0.5× TBE for 30 min prior to visualization using a UV transilluminator. Image analysis was performed using ImageJ.

### Immunofluorescence microscopy

In a six-well plate, 1 × 10^6^ Vero cells were transfected using lipofectamine 2000 (Invitrogen) in 0.2 ml Opti-MEM (Gibco) with 200 fmol FLAG-NS5*^ZIKV^* plasmid and fixed at the indicated time points with 4% PFA for 15 min and washed with PBS. Fixed cells were permeabilized by treating with 0.2% (v/v) Triton X-100/PBS for 5 min at room temperature before washing three times with PBS and then blocked by incubation with 5% (w/v) milk in PBS/0.1%Tween-20. Primary antibody against FLAG (M2 Mouse, Sigma, F1804), and secondary antibody was coupled to Alexa-Fluor 488 (Invitrogen). Cell nuclei were stained using DAPI (Sigma, 1:10 000 in water). Immunofluorescence microscopy was performed on an EVOS FL Auto 2 (Invitrogen).

### Viral genome nanoluciferase translation measurement

Capped, *in vitro* transcribed ZIKV*^Nluc^* RNA (1.5 pmol) was electroporated into 3 × 10^6^ Vero cells suspended in 100 μl of Opti-MEM (Gibco) using a NEPA21 electroporator (Nepagene). Cells were seeded sub-confluently in 24-well plates and samples were harvested for luciferase assays and RT-qPCR analysis at the indicated time points by washing with phosphate-buffered saline and lysis in passive lysis buffer (Promega). Nanoluciferase activity was measured using the Nano-Glo luciferase assay system (Promega) by GloMax (Promega).

### Virus production and titration

The capped ZIKV*^mCherry^* RNA was transfected in Vero cells using lipofectamine 2000 (Invitrogen) in 0.2 ml Opti-MEM (Gibco). 24 h post-transfection the cells were washed and supplemented with fresh media and monitored daily until significant cytopathic effect was observed, at which point supernatants were harvested, centrifuged at 1000 × *g* for 10 min to remove cellular debris and aliquots stored at −80°C. Virus was then titred by plaque assay. 100 μl of tenfold serial dilutions of virus samples were added to a six-well plate containing a confluent monolayer of Vero cells and incubated for 1 h with shaking after every 15 min. The cells were overlaid with 1 ml of DMEM supplemented with 2% FBS and 1% final concentration of low melting agarose (Sigma). After 4–7 days of incubation, the cells were fixed with 2% formaldehyde and stained with toluidine blue. Plaques were counted and viral titres in plaque forming units per ml calculated.

### mCherry virus detection- fluorescence microscopy and flow cytometry

In a six-well plate, 1 × 10^6^ Vero cells were transfected using lipofectamine 2000 (Invitrogen) in 0.2 ml Opti-MEM (Gibco) with 200 fmol of N-terminal FLAG NS5*^ZIKV^* or empty vector prior to infection with ZIKV*^mCherry^* virus (MOI:10). The cells were incubated with virus for 45 min and then washed twice with PBS and once with DMEM to remove the unadsorbed virus. 2 ml of complete medium was added and cells were incubated for 72 h. For the 0 h post infection (HPI) sample, cells were washed three times with PBS to remove the unadsorbed virus and harvested directly by treating with Accutase (BioLegend), washed again and resuspended in PBS. Cells were fixed with 2% paraformaldehyde (PFA) for 15 min. For other timepoints, cells were washed three times with PBS, harvested and fixed as above. For all time points after washing with PBS but before cell harvest, fluorescence microscopy was performed to monitor mCherry expression on an EVOS FL Auto 2 (Invitrogen). For quantification of mCherry signal, fixed cells were resuspended in PBS and analysed by flow cytometry using a BD Biosciences LSR Fortessa analyser and data was analysed using FACSDIVA software (BD Biosciences). A minimum of 10 000 cells were collected and analysed for each sample in three independent experiments.

### Puromycylation assay

In a six-well plate, 1 × 10^6^ Vero cells were transfected with 500 fmol NS5 expression plasmids or empty pCDNA3.1, using lipofectamine 2000 (Invitrogen) in 0.2 ml Opti-MEM (Gibco). After 1 h, cells were overlaid with 0.8 ml antibiotic-free DMEM then, after 7 h, 1 ml of media was added containing 10 μg/ml puromycin (5 μg/ml final concentration). Cells were harvested after 2 h in passive lysis buffer (Promega) for analysis by immunoblotting. Membranes were blocked in 5% milk PBST before probing with anti-puromycin (kindly shared by Prof. Ian Goodfellow, University of Cambridge—1:20 in Odyssey blocking buffer, LiCor), then anti-tubulin (1:1000 in 5% BSA PBST, Abcam ab-6046), visualized using IRDye 800CW goat anti-mouse and IRDye 680RD goat anti-rat secondary antibodies (LiCor), respectively. Protein signals were detected on an Odyssey CLx Imaging System (Li-Cor). Puromycylation signal was quantified using ImageJ.

## RESULTS

### ZIKV and DENV NS5 inhibit translation

To investigate the effect of NS5 on viral translation, we bacterially expressed and purified His-tagged full-length ZIKV NS5 (NS5*^ZIKV^*) (Supplementary Figure S1) and confirmed specific interaction with ZIKV SLA using an electrophoretic mobility shift assay (EMSA). NS5*^ZIKV^* bound an RNA fragment containing the first 359 nt of the ZIKV genomic RNA, encompassing the 5′ UTR and first 252 nt of the capsid sequence (ZIKV*^5^′^utr+^*), with nanomolar affinity (Figure 1D, upper panel). This affinity is consistent with that previously reported for DENV NS5 and DENV SLA (14). In contrast, NS5*^ZIKV^* failed to shift an RNA (Figure 1D, middle panel) mutated so as to destabilize SLA (ZIKV*^5^′^utr+^*-DS), as shown in Figure 1B. We next examined the impact of the NS5*^ZIKV^*/SLA interaction on translation *in vitro*. An *in vitro* transcribed and capped mRNA, comprising the first 158 nt of the ZIKV genomic RNA (contains SLA, SLB, cHP and 5′CS) followed by firefly luciferase (Fluc) in frame with the authentic viral translation start site and the complete ZIKV 3′ UTR (ZIKV*^Fluc^*, Figure 1E top schematic), was incubated with NS5*^ZIKV^* in a translation-competent Vero cell lysate. Translation was monitored by measuring luminescence from the luciferase reporter and normalized to the no polymerase control reaction. Both wildtype NS5*^ZIKV^*, and a mutant bearing a G_664_DD → G_664_AA substitution (NS5*^ZIKV^*-GAA) that disrupts the polymerase active site, reduced translation of ZIKV*^Fluc^* RNA (by 64% and 66%, respectively; Figure 1E). In contrast, addition of either protein failed to inhibit translation of the ZIKV*^Fluc^*-DS RNA in which SLA is mutated (as in Figure 1B), suggesting that the interaction between NS5 and SLA, but not the polymerase activity, is important for the translation inhibition observed. NS5*^ZIKV^* inhibited ZIKV*^Fluc^* RNA translation in rabbit reticulocyte lysate (RRL) to the same extent as the Vero lysate (Figure 1F) and so RRL was used for subsequent experiments.

We next examined if the translation inhibitory effect of NS5 is conserved in different flaviviruses. We expressed and purified His-tagged full-length DENV NS5 (NS5*^DENV^*) (Supplementary Figure S1) and confirmed its ability to bind to DENV RNA in an EMSA experiment (Figure 1G). Like NS5*^ZIKV^*, NS5*^DENV^* bound DENV*^5^′^utr+^* RNA with low nanomolar affinity (Figure 1G, upper panel) but did not bind a mutated version of DENV*^5^′^utr+^* (Figure 1G, middle panel) in which destabilizing mutations were introduced in SLA (DENV*^5^′^utr+^*-DS) as shown in Figure 1C. NS5*^DENV^* inhibited translation of a reporter RNA bearing the DENV SLA, SLB and cHP with an in frame Fluc reporter and the full DENV 3′ UTR (DENV*^Fluc^*, Figure 1H, upper schematic) but not a SLA destabilized mutant RNA (DENV*^Fluc^*-DS).

In EMSA experiments, NS5*^ZIKV^* only weakly associated with a short RNA fragment containing the 5′ UTR of human globin (Glob*^5^′^utr+^*) at the highest concentrations tested (Figure 1D, lower panel), while no interaction between NS5*^DENV^* and Glob*^5^′^utr+^* was detected (Figure 1G, lower panel). Consistently, NS5*^ZIKV^* and NS5*^DENV^* had a smaller inhibitory effect on the translation of a reporter RNA bearing the 5′ and 3′ UTRs of human globin (Glob*^Fluc^*, Figure 1F, upper schematic) than on the corresponding viral 5′ UTR containing Fluc reporter RNA (Figure 1F and H). The weak but significant inhibition of Glob*^Fluc^* translation in the presence of NS5*^ZIKV^* (Figure 1H) may result from addition of high concentrations of an RNA-binding protein to the reaction mixture, although this effect was not observed in the presence of NS5*^DENV^* (Figure 1H). These results demonstrate that NS5 can specifically inhibit translation in a manner consistent with its ability to bind SLA RNA.

### NS5 inhibits translation at the initiation stage

**Figure 2.**
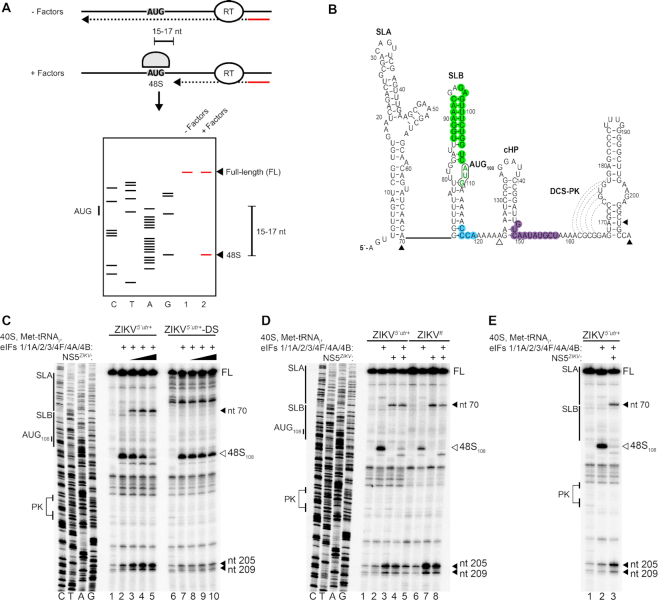
NS5*^ZIKV^* inhibits translation at the initiation stage. (**A**) Schematic of the *in vitro* reconstitution and toeprinting assay described in the text (from 6). (**B**) Nucleotide sequence and secondary structure of the ZIKV 5′ region. The start codon AUG_108_, (green font and black outline), UAR (green), DAR (blue) and 5′ CS (purple) are highlighted. Dotted lines indicate base pairing in the downstream of CS pseudoknot (DCS-PK). (**C–E**) Toeprinting analysis of 48S complex assembly on (C) capped ZIKV*^5^′^utr+^* or ZIKV*^5^′^utr+^*-DS RNA, (D) capped ZIKV*^5^′^utr+^* or full-length (ZIKV*^fl^*) RNA and (E) capped ZIKV*^5^′^utr+^*, in the presence of the indicated factors. The start codon and/or structural elements are labelled on the left. Toeprints caused by 48S complex assembly (open arrowhead) or NS5*^ZIKV^* binding (closed arrowhead) are marked on the right and also indicated in (B). NS5*^ZIKV^* was preincubated with the RNA before addition to the reconstitution reaction (C and D) or added to the reconstitution reaction at the same time as the translation factors (E). (C) NS5*^ZIKV^* was included at 64 nM (lanes 3 and 8), 128 nM (lanes 4 and 9) or 256 nM (lanes 5 and 10). (D) NS5*^ZIKV^* was included at 256 nM in the indicated lanes. FL, full length.

As NS5 binds to the highly conserved SLA at the 5′ extremity of the flaviviral genomic RNA, we hypothesized that it may interfere with translation factor recruitment and inhibit translation at the initiation stage. We recently reported an *in vitro* reconstitution method for examining flavivirus cap-dependent translation initiation (6). Using this system (39), individually purified small ribosomal subunits, eukaryotic translation initiation factors (eIFs) and initiator tRNA are combined to study the role of specific factors in regulating translation of a particular RNA. During translation initiation the 40S ribosomal subunit and associated eIFs are recruited to the 5′ cap of the mRNA (40). After ribosomal scanning of the 5′ UTR and translation start site selection, a stable 48S complex is assembled at the initiation site. Following start codon recognition, the 48S complex is poised for 60S ribosomal subunit joining and translation initiation. 48S complex assembly in the *in vitro* reconstitution system is monitored by detection of truncated cDNA products on a denaturing PAGE gel caused by the inhibition of reverse transcriptase (RT). The position of RT stops on the RNA are determined by comparison to a Sanger sequencing reaction. 48S complex assembly results in the appearance of truncated cDNA products +15–17 nt downstream of the start codon (shown schematically in Figure 2A). The secondary structure of the ZIKV 5′ region is shown in Figure 2B.

Addition of small ribosomal subunits, the canonical eIFs (1,1A, 2, 3, 4F, 4A, 4B) and initiator tRNA_i_ to ZIKV*^5^′^utr+^* resulted in a strong RT stop +16–17 nt downstream of AUG_108_, the start codon of the long viral-RNA open reading frame, characteristic of 48S complex assembly at this site (Figure 2C, compare lanes 1 and 2). Preincubation of ZIKV*^5^′^utr+^* RNA with NS5*^ZIKV^* before the addition of eIFs led to a dose dependent decrease in 48S complex assembly (Figure 2C, compare lane 2 to lanes 3–5). As a control addition of the eIF4A inhibitor hippuristanol caused a similar disappearance of the 48S dependent RT stop (Supplementary Figure S2). In contrast, preincubation of the SLA destabilized ZIKV*^5^′^utr+^*-DS RNA with the same concentrations of NS5*^ZIKV^* had a much weaker inhibitory effect on 48S complex assembly (Figure 2C, compare lane 7 to lanes 8–10), consistent with our *in vitro* translation data (Figure 1E). As apparent in Figure 2C, there is a difference in the RT arrest pattern in the region corresponding to SLA between the wild type and destabilized SLA RNAs providing evidence of a change in the RNA structure. This change is evident in the RNA only lanes as well as lanes containing translation factors and/or NS5. In contrast, no changes in the RT arrest pattern was observed in the rest of the RNA indicating that gross structural changes introduced by the SLA destabilizing mutations were restricted to the SLA region. NS5*^ZIKV^* preincubation also inhibited 48S complex formation on capped, full-length genomic ZIKV RNA (Figure 2D, compare lanes 6 and 8). To determine if NS5*^ZIKV^* could directly compete with the translation initiation machinery we performed the *in vitro* reconstitution experiment without prior incubation of the polymerase and the template RNA. As shown in Figure 2E, NS5*^ZIKV^* efficiently inhibits 48S complex assembly on ZIKV*^5^′^utr+^* RNA without prior incubation.

Inclusion of NS5*^ZIKV^* to the reconstitution system caused increased RT arrest at two positions independently of the translation components (Figure 2D, compare lane 1 to lanes 3 and 4). The first, at nt 71, is adjacent to the 3′ boundary of SLA, and the second at nt 205/209 occurs at the 3′ boundary of the first stem following the cHP in the capsid coding region. This stem forms part of the downstream of 5′ CS pseudoknot (DCS-PK) (Figure 2C, compare lane 2 to lanes 3–5). Disruption of the DCS-PK was previously reported to affect genome circularization and replication in DENV (41). Our data are consistent with previous results from WNV (15), indicating that a second binding site is conserved between at least some flaviviruses. Interestingly, competition EMSAs with a truncated WNV 5′ region lacking SLA indicated that the second site of NS5 interaction was of lower affinity as it could not outcompete binding to a SLA-containing RNA (15). Consistent with this, NS5*^ZIKV^* did not induce RT arrest at nucleotides 205/209 in the ZIKV*^5^′^utr+^* RNA in which the higher affinity SLA binding site was mutated (Figure 2C, compare lanes 5 and 10).

Although the mode of interaction between NS5 and SLA remains to be fully elucidated, previous studies have demonstrated that differing regions of NS5 contribute to interaction with SLA depending on which virus is being examined. For example, the polymerase domain of NS5*^DENV^* was reported as sufficient to bind SLA RNA in EMSA studies (14,21) whereas the polymerase domain of NS5 from WNV was not sufficient to interact with SLA (15). We generated a truncated His-tagged version of ZIKV NS5, NS5*^ZIKV-Pol^* containing amino acids 270–900, that includes the polymerase domain but lacks the methyl transferase domain. Consistent with the findings from WNV (15), the polymerase domain of NS5*^ZIKV^* did not associate with SLA containing RNA as examined by EMSA (Supplementary Figure S3A). NS5*^ZIKV-Pol^* did not inhibit 48S complex assembly when included in the *in vitro* reconstitution system and also failed to induce the appearance of the NS5*^ZIKV^* dependent RT arrest observed with the full-length protein (Supplementary Figure S3C). These results are consistent with a requirement for NS5 RNA binding to inhibit translation.

### NS5 inhibits translation of the viral genome in cells

**Figure 3.**
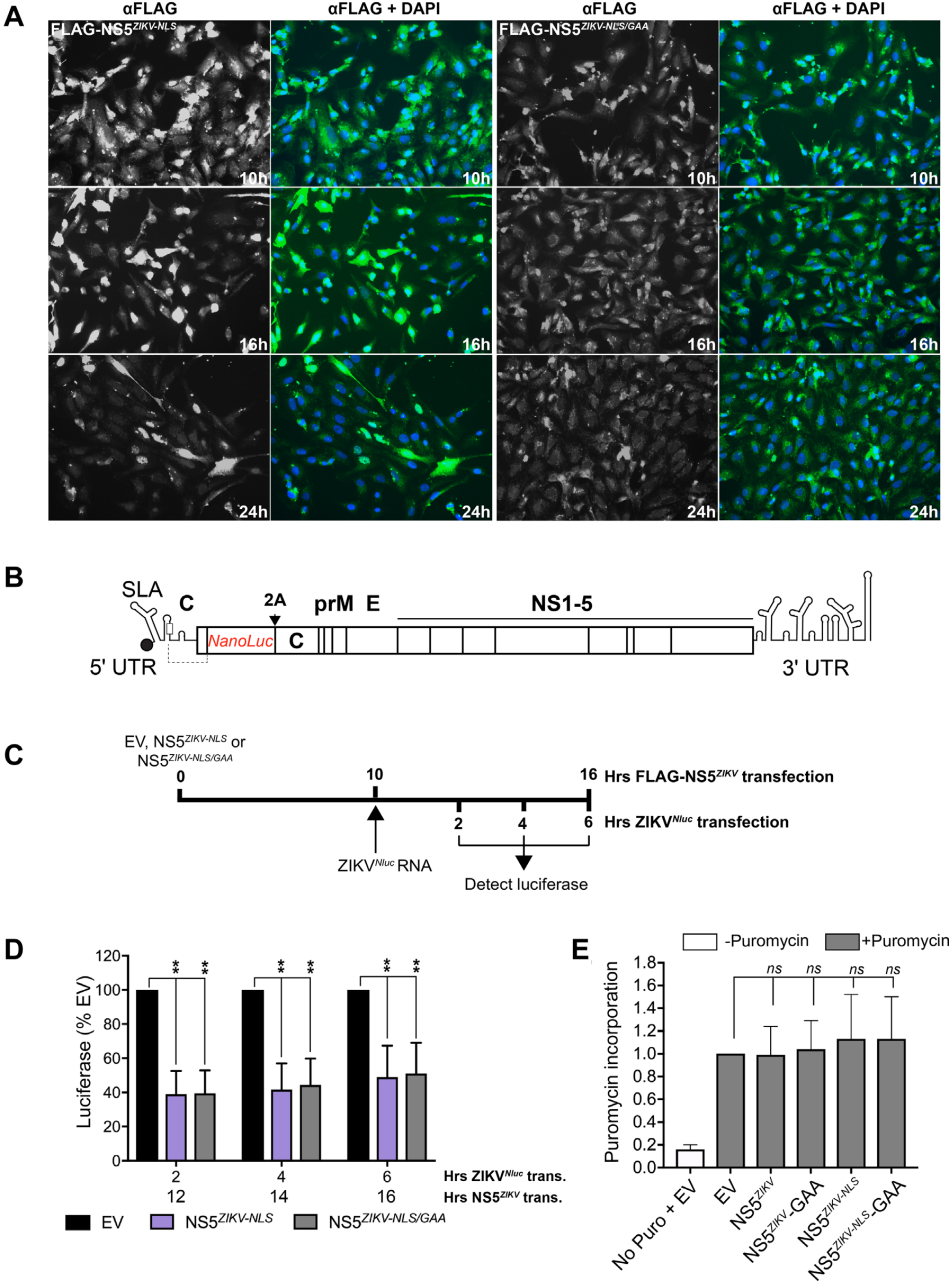
NS5*^ZIKV^* inhibits viral genomic RNA translation in cells. (**A**) Expression of FLAG-tagged NS5*^ZIKV^* was detected by immunofluorescence using an anti-FLAG antibody over a 24 h period following transfection of Vero cells. For each FLAG-NS5*^ZIKV^* expression construct tested anti-FLAG is shown in greyscale on the left while nuclei are stained blue with DAPI and anti-FLAG is green in the associated panel to the right. (**B**) Schematic of the reporter ZIKV bearing nanoluciferase (Nluc) (32). The duplicate capsid sequence is separated from the marker with a 2A StopGo sequence (indicated with 2A). (**C**) Experimental strategy used for D. (**D**) Vero cells were electroporated with capped, *in vitro* transcribed ZIKV*^Nluc^* marker RNA 10 h after transfection with the indicated FLAG-NS5*^ZIKV^* or empty vector (EV). Luciferase signal was quantified at the indicated time points. Values are normalized to the EV control at each time point and are mean ± SEM from three independent experiments. Time post NS5*^ZIKV^* transfection (*trans*.) and time post electroporation (*elec*.) of ZIKV*^Nluc^* RNA is included on the X-axis for clarity. Statistical significance was determined by a Student's unpaired *t*-test with significant values indicated with an asterisk. *P< 0.05, **P< 0.01. (**E**) Vero cells were transfected with empty vector (EV) or the indicated wild type or mutant FLAG-NS5*^ZIKV^* expressing plasmids and after 10 h puromycin was added as indicated and the cells incubated for a further 2 h. Protein lysates were analysed by western blotting using an anti-puromycin antibody. The signal was quantified for each lane and normalized to that for tubulin which was used as a loading control. A representative blot is shown in Supplementary Figure S4. Data are mean ± SEM from three independent experiments. Wildtype and mutant NS5 data were compared to EV by a Student's unpaired *t*-test and no significant change was observed.

To test the translation inhibitory activity of NS5 in cells we generated an N-terminally FLAG-tagged NS5*^ZIKV^* (FLAG-NS5*^ZIKV^*) expression construct and examined its effect on translation of a viral reporter RNA. To promote cytoplasmic retention of our overexpressed NS5 so that it could encounter the transfected reporter RNA, we introduced point mutations in the NLS (42) of NS5*^ZIKV^* (Lys390 and Arg393 to Ala) to generate FLAG-NS5*^ZIKV-NLS^*. In this background, we also mutated the active site G_664_DD → G_664_AA to minimize aberrant RNA replication activity in the FLAG-NS5*^ZIKV-NLS/GAA^* construct. Expression of FLAG-NS5*^ZIKV-NLS^* and FLAG-NS5*^ZIKV-NLS/GAA^* in Vero cells was determined by immunofluorescence following staining with an anti-FLAG antibody over the time course shown in Figure 3A. At 10 h post transfection, accumulation of NS5 in the cytoplasm was clearly visible and so we chose this time point for further experiments.

We investigated the impact of NS5 overexpression on translation in Vero cells of an *in vitro* transcribed and capped full-length ZIKV genomic RNA that contains a nanoluciferase reporter (ZIKV*^Nluc^*), shown schematically in Figure 3B. We transfected Vero cells with FLAG-NS5*^ZIKV-NLS^* or NS5*^ZIKV-NLS/GAA^* and 10 hours later introduced the ZIKV*^Nluc^* RNA by electroporation (timeline of experiment shown in Figure 3C). Translation of the viral genomic RNA, as measured by nanoluciferase production, was decreased by 60% in the FLAG-NS5*^ZIKV^* expressing cells when compared to the empty vector control (Figure 3D). The luciferase signal remained lower for 6 hours post transfection (Figure 3D) after which point the control luciferase signal began to decrease (data not shown) in line with our previous results (6). The presence of NS5 did not affect global protein synthesis in the cell as measured by puromycin incorporation (Figure 3E and Supplementary Figure S4). Together, our results identify a previously undescribed role for flavivirus NS5 as a regulator of virus translation.

### Overexpression of NS5*^ZIKV^* inhibits viral replication

**Figure 4.**
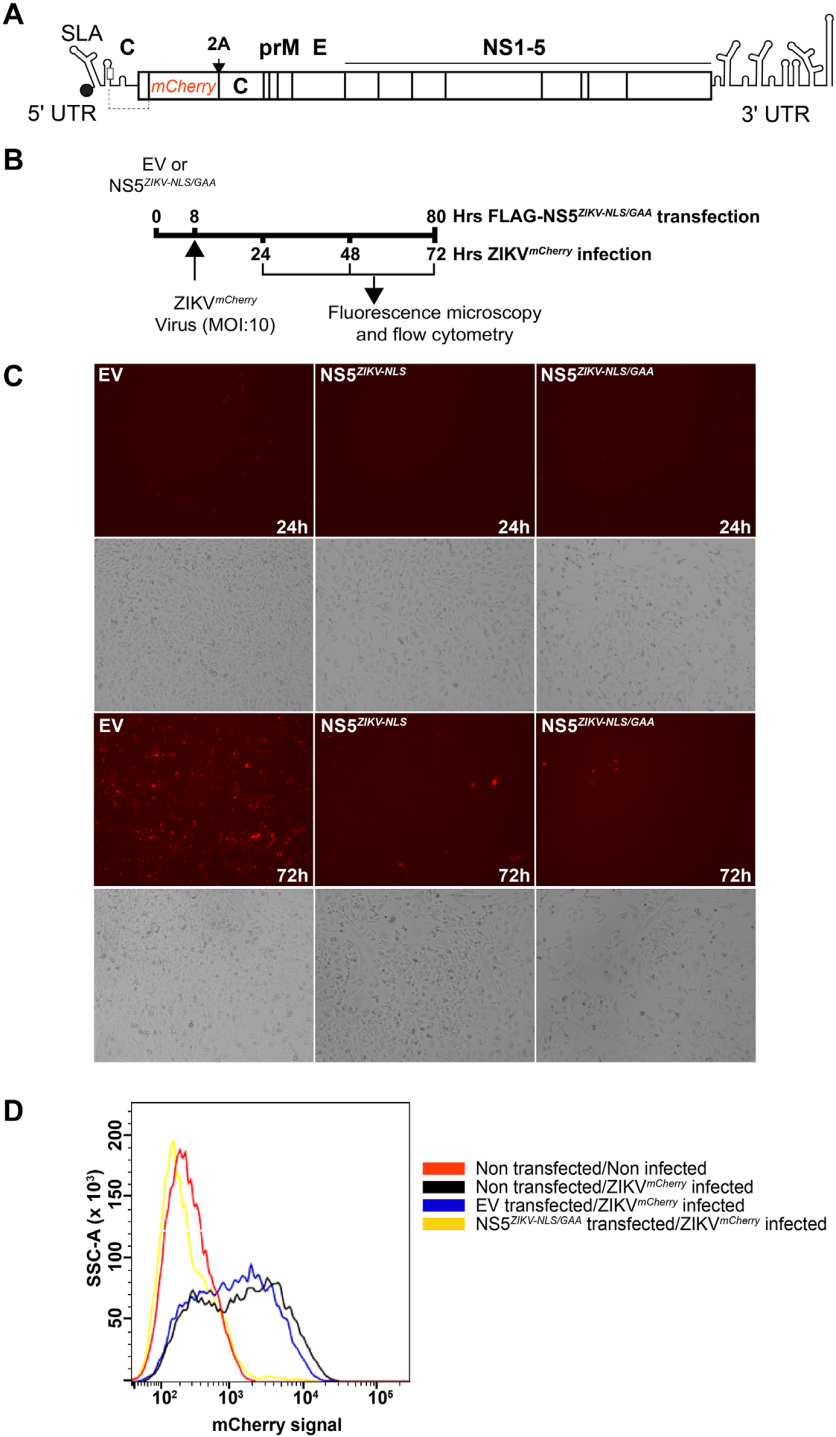
NS5*^ZIKV^* pre-expression disrupts viral replication. (**A**) Schematic of the reporter ZIKV bearing nanoluciferase (Nluc) (32). The duplicate capsid sequence is separated from the marker with a 2A StopGo sequence. (**B**) Experimental strategy used for C–E. (**C** and **D**) Vero cells were infected with mCherry reporter virus (MOI:10) 8 h after transfection with FLAG-NS5*^ZIKV-NLS/GAA^* or empty vector (EV). (C) mCherry signal was detected directly using a fluorescence microscope at the indicated time points. The bright field view is shown below each fluorescence image. (D) Cells were fixed and analysed by flow cytometry 72 hours after infection. See also Supplementary Figure S5.

As our data demonstrated that NS5 inhibits viral translation we next examined the effect of NS5 expression on viral replication. We generated high titre stocks of ZIKV containing an mCherry reporter (ZIKV*^mCherry^*, shown schematically in Figure 4A). The production of this tagged ZIKV has been previously reported (32). Using a similar strategy to our nanoluciferase experiments we first overexpressed FLAG-NS5*^ZIKV-NLS^* or FLAG-NS5*^ZIKV-NLS/GAA^* for 8 hours in Vero cells before infecting with a high multiplicity of infection (MOI:10) of ZIKV*^mCherry^* (Figure 4B). Viral replication was then monitored by detection of the mCherry signal on a fluorescence microscope. Transfection of either FLAG-NS5*^ZIKV^* constructs resulted in a marked delay in replication of the mCherry tagged ZIKV as compared to transfection with empty vector, as evidenced by the lower fluorescence signal detected at each time point analysed (Figure 4C). The NS5-induced delay in ZIKV replication observed in our fluorescence microscopy experiments was also confirmed by flow cytometry analysis. The number of mCherry positive cells increased at each time point examined following ZIKV*^mCherry^* infection of non-transfected or empty vector control plasmid transfected cells (Supplementary Figure S5 and Table S1, 72 hour time point shown in Figure 4D). In contrast, transfection of FLAG-NS5*^ZIKV-NLS^* or FLAG-NS5*^ZIKV-NLS/GAA^* prior to infection resulted in a decrease in the number of mCherry positive cells at each time point examined (72 h time point shown in Figure 4D, see also Supplementary Figure S5 and Table S1). Our finding that the presence of NS5 before infection impairs ZIKV replication is consistent with its ability to inhibit translation of the viral genome and has important implications for establishing *trans*-replicase plasmid-based systems for examining flavivirus replication as recently developed for other positive sense RNA viruses (43).

## DISCUSSION

Viruses with limited genome lengths frequently encode proteins with multiple functions. RNA viruses, in particular, also evolve complex genome structures that regulate various stages of the replication cycle, thus maximizing the usage of coding and non-coding regions of viral RNA. Here we have shown that, along with serving as the well described replication promoter (14), the interaction of the flavivirus NS5 protein with the structured 5′ UTR serves a secondary role as a mechanism to control translation of the viral genome.

Genome circularization through hybridization of complementary RNA sequences in the 5′ and 3′ regions is essential for flavivirus replication (44). It was previously reported that NS5 binds more efficiently to SLA in the linear form of a DENV4 minigenome RNA *in vitro* (45), although the difference detected in EMSA experiments was small. We recently demonstrated that the circularized, replication-competent form of the viral RNA is translated less efficiently than the linear form due to structural rearrangements that occur around the translation start site (6), providing a mechanism to maintain a ribosome clear template for replication. Although the difference in affinity was small, we also detected enhanced binding of ZIKV NS5 to the linear form of ZIKV RNA (Supplementary Figure S6), supporting the hypothesis that NS5 is recruited to RNA in the linear conformation (45). This is consistent with NS5 binding to and inhibiting protein synthesis on the translation-competent form of the genomic RNA. As multiple viral proteins are involved in establishing a replication complex (46) and have been reported to promote genome circularization (47,48), locating the NS5 RdRp at the C-terminus of the large open reading frame, and sequestration within the viral polyprotein, may delay initiation of replication until other viral factors have been synthesized. Future work will investigate the contribution of NS5 translation inhibition to the temporal switch to signal that sufficient viral proteins have been synthesized to initiate replication.

**Figure 5.**
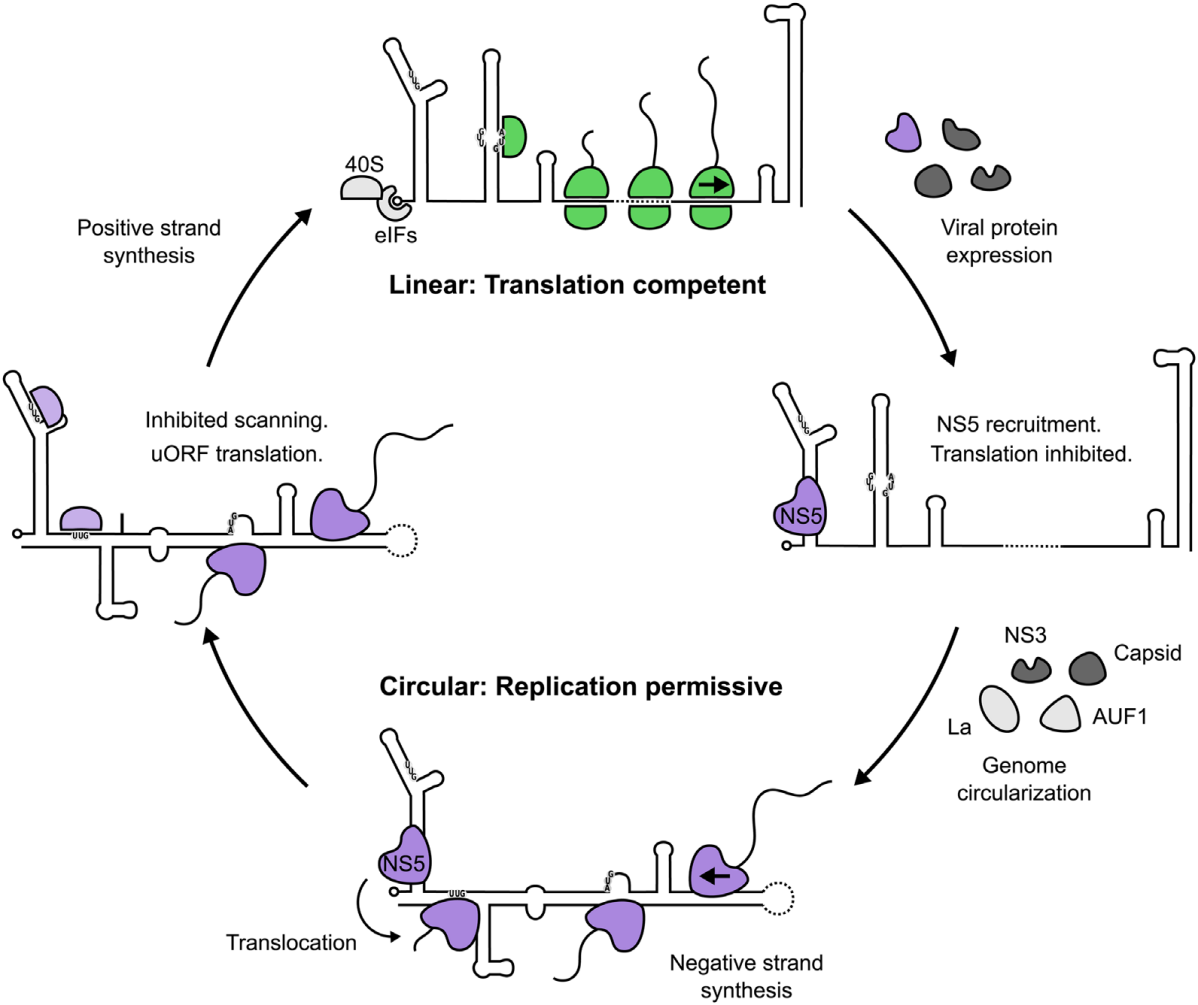
Model of flavivirus genome translation regulation. Linear genomic RNA recruits eIFs and 40S ribosomal subunits in a cap-dependent manner (6). Secondary structure in the 5′ UTR is effectively resolved by eIFs and the scanning ribosome to allow efficient translation from the authentic initiation codon, leading to the accumulation of viral proteins. NS5 is subsequently recruited to SLA and antagonizes translation initiation, priming the RNA for replication. Following genome circularization promoted by viral and host factors (47,48,50,51,58), NS5 translocates to the 3′ end of the genome to begin negative strand synthesis. The circularized conformation of the viral genome acts as a barrier to prevent further ribosome loading by impeding scanning ribosomes (6), indicated by the red stop sign, thus keeping the replicating template free of ribosomes.

Our findings support a model for flavivirus replication (Figure 5) whereby the viral genome is efficiently translated after release into the cytoplasm in the linear form but, after sufficient NS5 is expressed, NS5 binds to the cap-proximal SLA structure and inhibits further translation of the viral genome. Subsequent circularization of the genome, promoted by viral and cellular factors (47–51), mediates transfer of NS5 to the viral genome 3′ end to initiate replication. It is possible that genome circularization depends upon ribosome clearance from the RNA template as, by analogy with bacteria, the ribosome has been described as a major helicase activity within the cell (52,53). As such, NS5 recruitment to the 5′ end, together with genome circularization (6), may provide distinct mechanisms for decreasing ribosome occupancy on the viral RNA by inhibiting translation initiation thus facilitating efficient genome replication.

**Figure 6.**
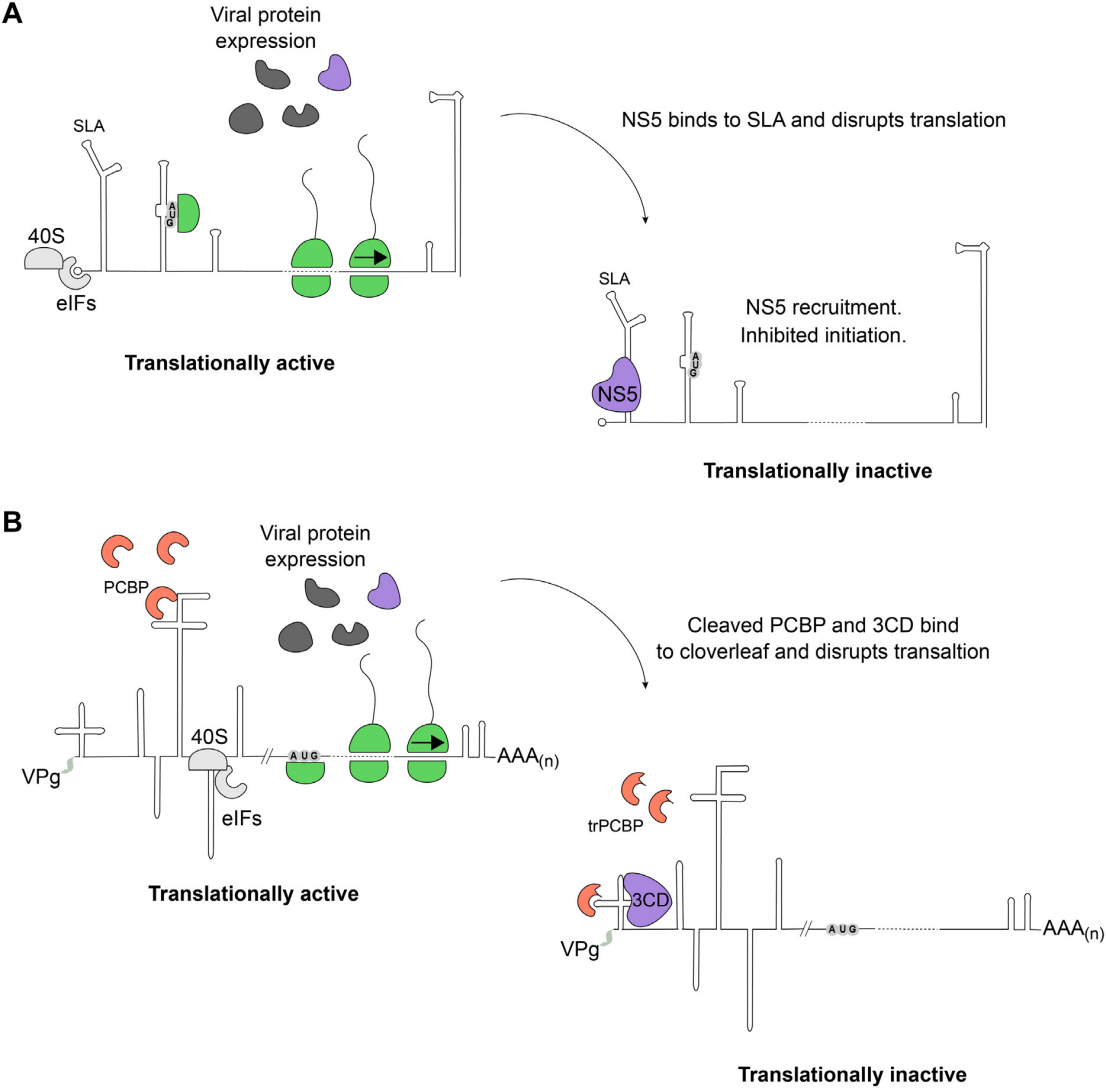
Model for conserved mechanisms of temporal translation control in positive strand RNA viruses. (**A**) Flavivirus RNA is translated in a cap-dependent manner following genome release into the cytoplasm. As the viral polymerase NS5 is expressed it binds to the stem loop A (SLA) replication promoter at the 5′ end of the genome preventing translation initiation. (**B**) Poliovirus (PV) translation, driven by an internal ribosomal entry site (IRES), occurs after release of the genome to the cytoplasm. The cellular polyC-binding protein (PCBP) is required for PV IRES activity (57). As the viral protease/polymerase precursor 3CD is expressed, it can subsequently bind to the 5′ cloverleaf structure and disrupt IRES dependent translation. PCBP is also cleaved by the viral protease so it can no longer promote IRES translation. In both cases, viral translation arrest clears the template for efficient replication.

This proposed role for NS5 in temporally regulating translation of 5′ capped flaviviral RNA is reminiscent of the similar role postulated for the picornaviral 3CD protease/polymerase precursor protein during poliovirus (PV) replication (54,55) (Figure 6). Like flaviviruses, picornaviruses possess a single-stranded positive sense RNA genome, translation of which must be inhibited to promote efficient replication (54). Binding of 3CD to the cloverleaf structure at the 5′ of the PV genomic RNA was reported to regulate PV translation (54) which is driven by a cap-independent, internal ribosomal entry site (56,57). Although the mechanism of PV 3CD-IRES translation control has not been fully elucidated, together with our results, these findings point to a conserved system of controlling the timing of the translation–replication switch in different positive-sense single-stranded RNA virus families exploiting the RNA-binding capabilities of the viral polymerase.

## Supplementary Material

gkaa242_Supplemental_File
