# Supplementary material for: The flavivirus polymerase NS5 regulates translation of viral genomic RNA
Source: Nucleic Acids Res. 2020 Apr 20;48(9):5081–93. doi: 10.1093/nar/gkaa242 (PMC7229856; doi:10.1093/nar/gkaa242)
Supplement: gkaa242_Supplemental_File [file gkaa242_supplemental_file.pdf]

Supplemental Information for:

The flavivirus polymerase NS5 regulates translation of viral genomic RNA.

Teodoro Fajardo, Jr[1†], Thomas J. Sanford[1†], Harriet V. Mears[1], Annika Jasper[1], Skye Storrie[1], Daniel S. Mansur[2], and Trevor R. Sweeney[1*].

1 Division of Virology, Department of Pathology, University of Cambridge, Addenbrooke's Hospital, Hills Road, Cambridge, UK.
2 Laboratory of Immunobiology, Department of Microbiology, Immunology and Parasitology, Universidade Federal de Santa Catarina, Florianópolis, Brazil.

* To whom correspondence may be addressed, email: ts629@cam.ac.uk, phone: +44(0)1223336072, fax: +44 1223336926

† These authors contributed equally to this work.

Contains Figures S1-S6 and Table S1

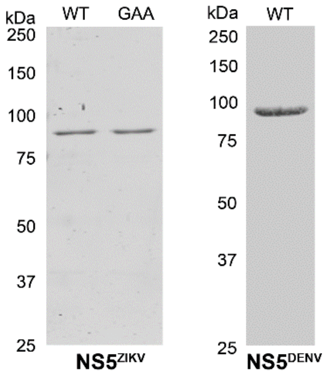

Figure S1. Purified NS5 proteins used in EMSA and translation assays
(**A**) Coomassie stained gel of purified His-tagged NS5^{ZIKV} (left panel) and NS5^{DENV} (right panel). WT, wild type. GAA, G_{664}DD → G_{664}AA polymerase active site mutated NS5.

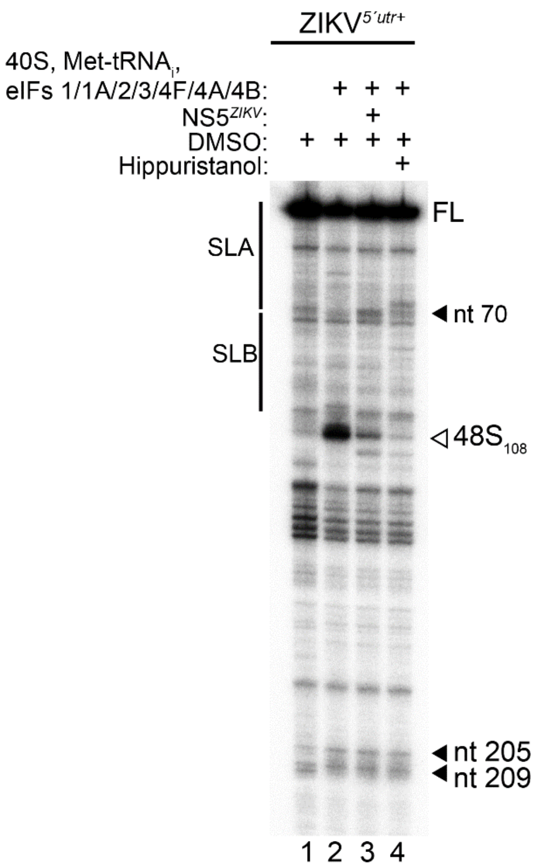

Figure S2. Comparison of effect of NS5 and hippuristanol on 48S complex assembly in the in vitro reconstitution assay.
Toeprinting analysis of 48S complex assembly on capped ZIKV$^{5'utr+}$ in the presence of the indicated factors. The structural elements are labelled on the left. Toeprints caused by 48S complex assembly are marked on the right (open arrowhead). NS5^{ZIKV} was added to the reconstitution reaction at the same time as the translation factors. NS5^{ZIKV} was included at 256 nM (lane 3). Hippuristanol (suspended in DMSO) was included at 5 µM final concentration (lane 4). FL, full length. The presence of DMSO reduced the intensity of the RT arrests induced by NS5^{ZIKV} binding (closed arrowheads) but the impact of NS5^{ZIKV} on 48S complex assembly was unaffected (compare lanes 2 and 3).

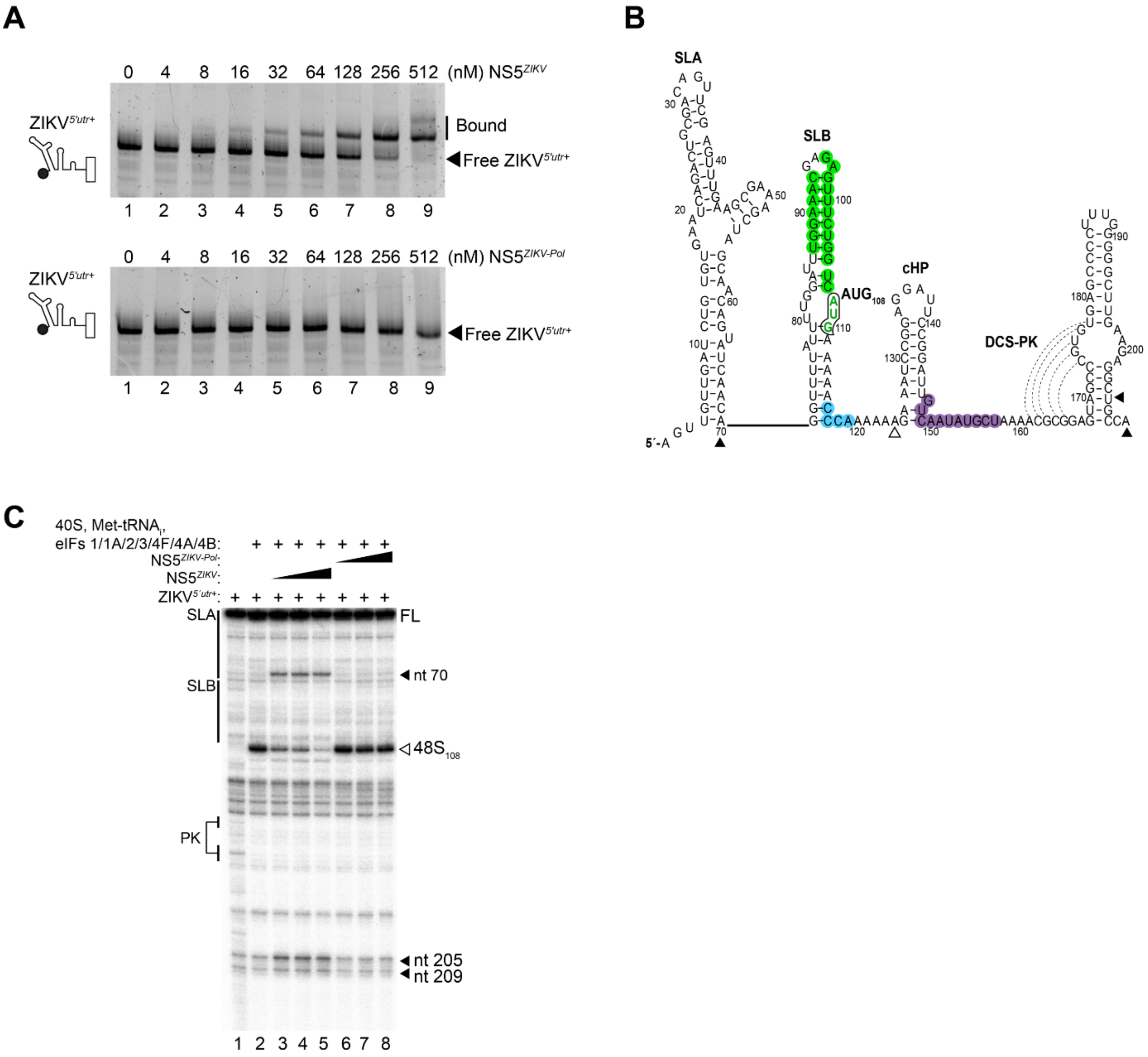

Figure S3. Comparison of full length and the polymerase only domain of $NS5^{ZIKV}$ in EMSA and *in vitro* reconstitution assays.
(**A**) EMSA of the first 359 nt of wildtype $ZIKV^{5'utr+}$ (shown schematically on left) with increasing concentrations of $NS5^{ZIKV}$ full-length (upper panel) or amino acids 270-900 comprising the polymerase domain only ($NS5^{ZIKV\text{-}Pol}$, lower panel). Free and bound RNA are indicated. (**B**) Nucleotide sequence and secondary structure of the ZIKV 5′ region. The start codon AUG_{108}, (green font and black outline), UAR (green), DAR (blue) and 5′ CS (purple) are highlighted. Dotted lines indicate base pairing in the downstream of CS pseudoknot (DCS-PK). (**C**) Toeprinting analysis of 48S complex assembly on capped $ZIKV^{5'utr+}$ in the presence of the indicated translation factors and either full-length or the polymerase only domain of $NS5^{ZIKV}$. The RNA structural elements are labelled on the left. Toeprints caused by 48S complex assembly (open arrowhead) or $NS5^{ZIKV}$ binding (closed arrowhead) are marked on the right and also indicated in B. Full-length $NS5^{ZIKV}$ or $NS5^{ZIKV\text{-}Pol}$ was added to the reconstitution reaction at the same time as the translation factors. $NS5^{ZIKV}$ or $NS5^{ZIKV\text{-}Pol}$ was included at 64 nM (lanes 3 and 6), 128 nM (lanes 4 and 7) or 256 nM (lanes 5 and 8). FL, full length.

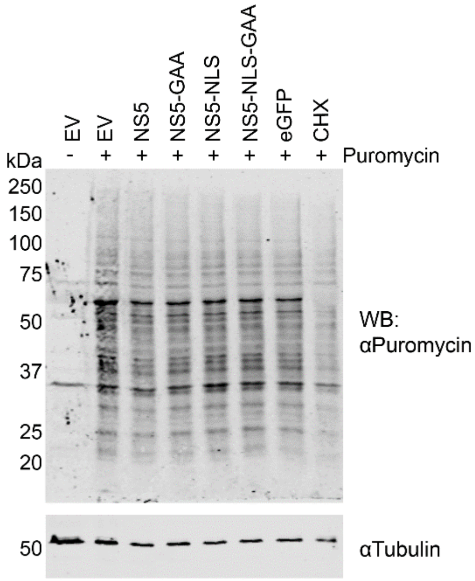

Figure S4. Representative gel from puromycylation experiment. Vero cells were transfected with the indicated NS5 expression plasmids or empty vector (EV) and after 8 hours overlaid with an equal volume of media containing 10 ug/mL puromycin (5 ug/mL final concentration). Cells were harvested after a further 2 hours and lysates examined by western blotting using the indicated antibodies. Gel is representative of three independent experiments. eGFP; green fluorescent protein. CHX; cycloheximide.

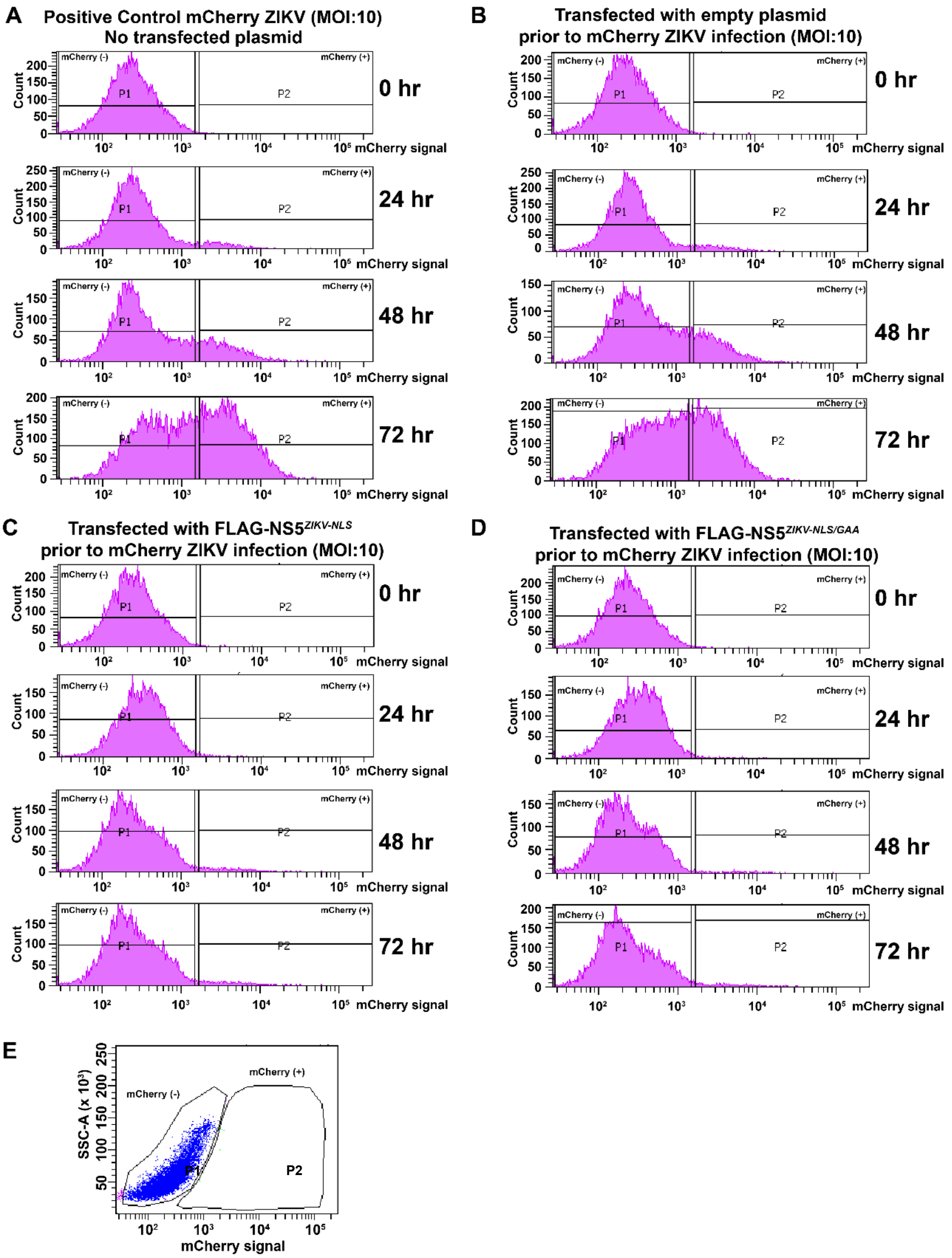

Figure S5. Flow cytometry analysis of mCherry ZIKV infection

Vero cells were transfected with empty vector (EV) or a plasmid expressing FLAG-tagged NS5$^{ZIKVNLS/GAA}$ (NS5^{ZIKV}) and infected with mCherry after 8 hrs. (**A-D**) Histograms for cells treated as indicated at the top of each panel were fixed at the indicated time points and analysed by flow cytometry. (**E**) shows the gating strategy used, set for the positive control mCherry ZIKV infected Vero cell sample (no transfected plasmid) 0 hr post infection. Data are representative of three experiments. P1 is the non mCherry expressing population set using the non-infected cell sample. P2 is the mCherry expressing cell sample. The percentage cells in the mCherry negative and positive gates are shown in Table S1.

Table S1. Flow cytometry analysis of mCherry ZIKV infected cells

**Events = 10,000**	**0 hpi**		**24 hpi**		**48 hpi**		**72 hpi**	
	EV	NS5^{ZIKV}	EV	NS5^{ZIKV}	EV	NS5^{ZIKV}	EV	NS5^{ZIKV}
**Cells**	95.6%	94.8%	95.9%	83.6%	92.8%	83.1%	90.4%	88.8%
**Singlets**	98.8%	99.0%	99.2%	98.0%	98.9%	97.5%	98.5%	96.9%
**mCherry negative**	99.1%	99.1%	91.1%	98.8%	63.6%	97.1%	35.2%	95.9%
**mCherry positive**	0.2%	0.1%	8.2%	0.7%	34.0%	1.7%	62.4%	3.2%

EV- empty vector, cells were transfected with pcDNA3.1 plasmid and infected 8 hr later with mCherry ZIKV (MOI:10).

NS5^{ZIKV}- cells were transfected with pcDNA3.1 containing FLAG-tagged NS5$^{ZIKV-NLS/GAA}$ plasmid and infected 8 hr later with mCherry ZIKV (MOI:10).

Cells were fixed and sorted at the indicated timepoints. Data are representative of three independent experiments.

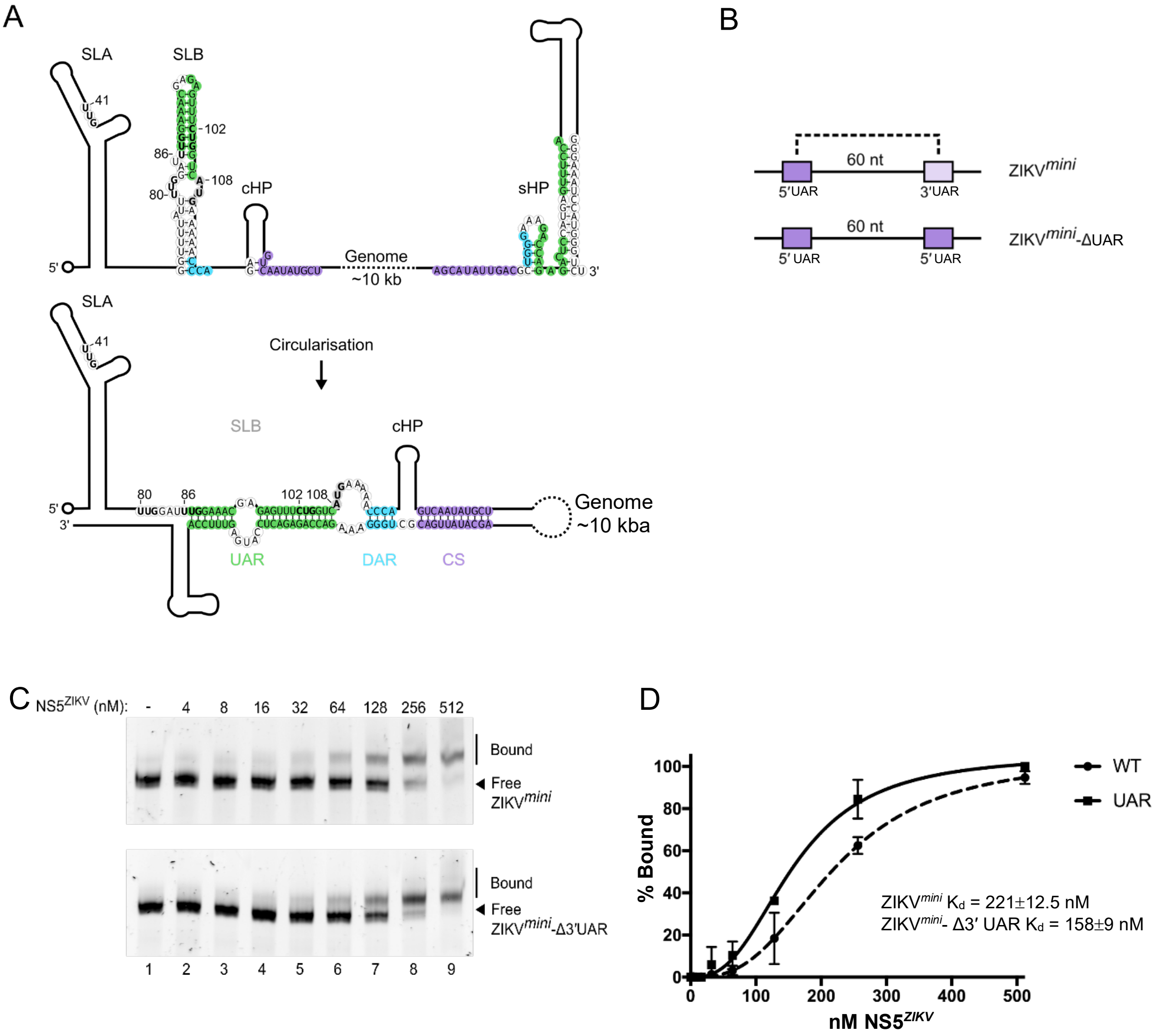

Figure S6. NS5^{ZIKV} binds the linear genome with a higher affinity than circularized genome.

(**A**) Schematic of the linear and circularized forms of the ZIKV genome. (**B**) Representation of the RNA examined. The ZIKV^{mini} RNA contains the first 157 nt and full 3′ UTR of ZIKV PE243 separated by 60 nt spacer sequence (6). This RNA was previously demonstrated to adopt the circularized conformation (6). To disrupt circularization potential a mutant RNA was generated (ZIKV^{mini}- Δ3′ UAR) in which the sequence in the 3′ UTR complementary to the 5′ UAR sequence was replaced by the 5′ UAR sequence. This RNA was previously demonstrated to adopt the linear conformation (6). The dotted line indicates a propensity to circularize. A and B are from (6). (**C**) EMSA analysis of ZIKV^{mini} and ZIKV^{mini}- Δ3′ UAR RNA and increasing concentrations of NS5^{ZIKV}. Free and bound RNA are indicated. (**D**) Analysis of the EMSA experiment shown in (C). Data are the mean of two independent experiments +/- SD and curves fitted by non-linear regression using GraphPad Prism software.
